# Association of Physical Function, Quantity, and Quality of the Quadriceps with Postoperative Physical Activity Before Total Knee Arthroplasty

**DOI:** 10.3390/jcm14010294

**Published:** 2025-01-06

**Authors:** Takaya Watabe, Takuya Sengoku, Goro Sakurai, Shinya Yoshida, Yuta Taniguchi

**Affiliations:** 1 Section of Rehabilitation, Kanazawa University Hospital 13-1 Takaranachi, Kanazawa 920-8641, Ishikawa, Japan; 2 Institute of Science & Engineering, Kanazawa University, Kakuma-Machi, Kanazawa 920-1192, Ishikawa, Japan; 3 Department of Orthopedic Surgery, Graduate School of Medicine Sciences, Kanazawa University, 13-1 Takaranachi, Kanazawa 920-8641, Ishikawa, Japan

**Keywords:** knee osteoarthritis, muscle quantity, muscle quality, physical activity, quadriceps femoris, total knee arthroplasty

## Abstract

**Background/Objectives:** This single-center cohort study investigated preoperative risk factors such as physical function, quantity, and quality of the quadriceps femoris for physical activity (PA) 1 year after total knee arthroplasty (TKA). **Methods:** This study included 204 patients with knee osteoarthritis who underwent TKA; they were divided into increased and decreased PA groups. Items with significant differences between the two groups in non-operative-side quadriceps strength, knee injury and osteoarthritis outcome scores (KOOS), Sport/Rec scores, operative-side cross-sectional area (CSA) of the vastus medialis (VM), and operative-side computed tomography attenuation values (CTV) of the vastus lateralis (VL) were fitted in the multiple logistic regression analysis. The cutoff value of the preoperative CSA of the VM required for PA to exceed the required points at 1 year postoperatively was calculated using the receiver operating characteristic (ROC) curve. **Results**: Multivariate logistic regression analysis showed that the non-operative-side quadriceps strength KOOS sport/rec, operative-side CSA of VM, and operative-side CTV of the VL were significantly associated with increased PA after TKA. The ROC-calculated cutoff value was 10.2 cm^2^. **Conclusions:** These results suggested that preoperative muscle quantity and quality, particularly in the VM, could play important roles in postoperative PA outcomes after TKA.

## 1. Introduction

Knee osteoarthritis (KOA) is a degenerative joint disease common in older persons and the most common cause of limited mobility in adults [1,2]. Pain and functional limitations caused by KOA negatively affect physical activity (PA) and quality of life (QOL) [3]. Total knee arthroplasty (TKA) aims to improve physical function and PA, enhance health-related QOL, and prolong healthy life expectancy [4,5,6]. Therefore, postoperative PA is an important outcome in patients undergoing TKA. However, approximately 10–33% of patients undergoing TKA continue to experience persistent pain and difficulty when performing daily activities [7]. A contributing factor to this is muscle weakness, which is ubiquitous before TKA and further amplified after it and linked to poor physical function and activity [8,9].

The quadriceps muscle controls knee extension and is the main active knee stabilizer; its correct function strongly influences patients’ return to PA after TKA [10]. KOA leads to quadriceps weakness, which may hasten osteoarthritis progression [11]. Mizner et al. found that preoperative quadriceps strength predicts functional performance assessed through the stair climb and timed “Up & Go” tests 1 year after TKA [12]. In addition, previous studies have reported that preoperative contralateral quadriceps strength predicted PA and walking speed after TKA [13,14]. Additionally, muscle evaluation using computed tomography (CT) has shown that the quadriceps cross-sectional area (CSA) on mid-thigh CT is a good predictor of whole-body skeletal muscle mass [15], and quadriceps CT attenuation values (CTV) on mid-thigh CT negatively correlated with PA [16]. Fat and muscle tissues could easily be distinguished using CT because fat has a negative CTV, whereas muscle has a positive CTV [17]. Infiltration of fats into the quadriceps has a more detrimental effect on PA and activities of daily living (ADL) than the loss of quadriceps muscle mass [16]. Therefore, preoperative decreases in quadriceps strength, CSA, and CTV might affect postoperative PA. However, the preoperative quantity and quality of the quadriceps, which could influence PA after TKA, remain unclear.

This study aimed to determine preoperative risk factors such as physical function, quantity, and quality of the quadriceps femoris for PA one year after TKA. It was hypothesized that if preoperative quadriceps strength, operative-side CSA, and CTV of the quadriceps decreased, then postoperative PA would decrease.

## 2. Methods

### 2.1. Study Design

**Figure 1:**
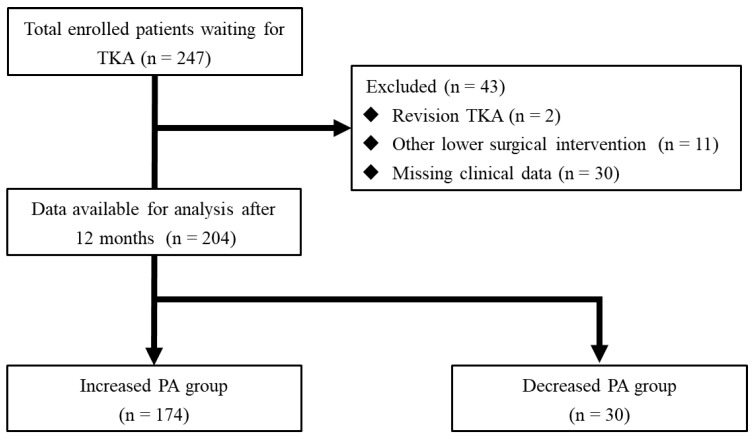
Flow chart of patient recruitment into this study.

This single-center cohort study included patients with KOA who underwent primary TKA. Between January 2019 and August 2023, 247 patients were consecutively enrolled in the participating hospitals (Figure 1). The exclusion criteria were revision TKA, severe cognitive deficits, speaking a primary language other than Japanese, lower limb surgical intervention, and a decline in patient participation. Based on these criteria, we excluded 43 patients; 204 were ultimately included in this study. The patients were divided into two groups based on an increased or decreased postoperative item number 4 on the Japanese version of the 2011 Knee Society Score (KSS) 1 year after the preoperative score was established. Written informed consent was obtained from all patients before participating in this study. Our institution’s Ethics Review Committee approved this study (113,786).

### 2.2. Surgical Technique and Rehabilitation Protocol

The TKA was performed by two orthopedic surgeons at the university hospital. All TKA procedures were performed using the subvastus and medial parapatellar approaches with the mechanical alignment method. Postoperative rehabilitation was started the day after surgery with full weight-bearing of the operative leg. The goal of rehabilitation was to obtain independent gait with a cane at 2 weeks postoperatively. The rehabilitation programs included exercises to improve knee flexion and extension angles, leg strength training, gait training, and exercises to achieve ADL motion.

### 2.3. Assessment Variables

The clinical parameters assessed included basic information (sex, age at the time of surgery, height, weight, body mass index, history of contralateral TKA, approach, Kellgren–Lawrence grade, 10 m gait time, range of motion (ROM) of knee flexion and extension, quadriceps muscle strength, one-leg standing time, and knee pain [numerical rating scale, NRS]); femorotibial angle; and knee injury and osteoarthritis outcome scores (KOOS). Preoperative skeletal muscle assessments included the CSA and CTV of the vastus medialis (VM), vastus lateralis (VL), vastus intermedius (VI), and rectus femoris (RF).

### 2.4. Physical Function Tests

We measured the 10 m gait time at a comfortable pace over a 14 m straight path with an additional 2 m at the beginning and end of the runway [18]. The time required for patients to achieve a 10 m walk was measured using a stopwatch. Passive ROM was measured using a goniometer (Toudaisiki Goniometer; OG Wellness Co., Ltd., Okayama, Japan). Quadriceps and hamstring strengths were measured using a handheld dynamometer (μTas F-1; ANIMA, Tokyo, Japan). Handheld dynamometer assessments were performed using a technique validated for measuring quadriceps strength [19]. The participants were seated with their hip and knee joints at 90° flexion, and the isometric knee force was measured twice. The highest recorded value, expressed in newtons, was used for analysis. The time until the participants lowered their raised leg (when the raised leg touched the floor due to an inability to maintain the one-legged stance) was measured twice, with a maximum time limit of 60 s. The better of the two measurements was used for the statistical analysis. The one-leg standing time was a clinical tool for assessing postural instability in a static position [20]. The NRS rated pain on a scale of 0–10, with 0 indicating no pain and 10 indicating the worst pain.

### 2.5. Patient-Reported Outcome Measures

The patients were asked to complete the Japanese version of the KSS twice: preoperatively and at 1 year follow-up [21]. The patients completed the KSS at the hospital. PA was measured preoperatively and at 1 year after TKA using item 4 (PA) on the KSS system from a 0 to 100-point scale (100 being the highest amount of PA) [22]. This scoring system was a 4-item assessment of symptoms, expectations, satisfaction, and PA of patients after TKA [22].

The patient-reported outcome measures collected in this study comprised the KOOS, a 42-item self-administered knee-specific questionnaire for assessing pain (nine items), symptoms (seven items), ADL (17 items), sports and recreation function (five items), and knee-related QOL (four items) as five separate subscales. The patients answered each item by marking one of five response options on a Likert scale. Scores ranging between 0 (extreme problems) and 100 (no problems) were calculated for each subscale [23].

### 2.6. Skeletal Muscle Assessment

**Figure 2:**
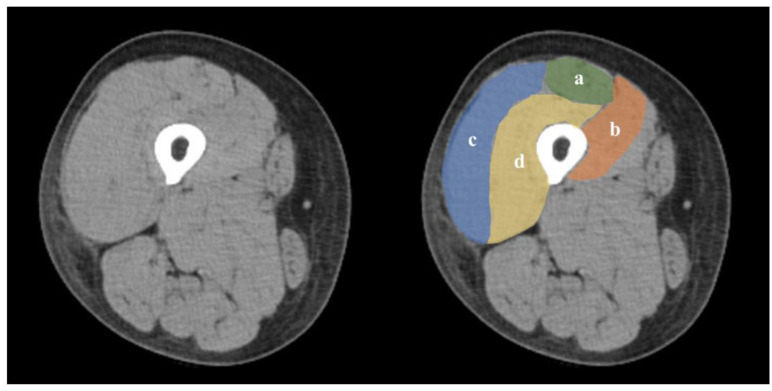
Measurement of quadriceps femoris using CT. Mid-thigh CT. a: Rectus femoris. b: Vastus medialis. c: Vastus lateralis. d: Vastus intermedius. CT, computed tomography.

We assessed muscle quantity and quality by measuring the CSA and evaluating the CTV, respectively, on cross-sectional CT imaging (Figure 2). These measurements were taken at the mid-thigh, specifically at the midpoint between the superior pole of the patella and inguinal crease (settings: 120 kV, 120 mA; rotation time: 1 s; and field of view: 233 mm) [24], using EV Insite image analysis software (PSP Corporation, Tokyo, Japan). In each patient, manual range selection was applied to the VM, VL, VI, and RF, after which CSA and CTV were calculated for each group [24]. CSA indicated muscle mass and intermuscular fat, whereas CTV provided a measure of muscle quality, reflecting the presence of intramuscular and intramyocellular lipids. The average CTV for muscles typically ranged between 40 and 100 Hounsfield Units (HU). Muscles with low HU values indicated a reduced muscle density (reduced muscle quality). A previous study reported the reliability and validity of this measurement as feasible [25].

### 2.7. Data Analyses

The power of this study was determined using G* Power 3.1.9.7 (Franz Paul, Kiel, Germany). A priori power analysis was used to calculate the sample size for the univariate analysis; the following results were obtained: 142 patients were needed based on effect size (d) of 0.50, an α level of 0.05, and a power of 0.80. In addition, a priori power was used to calculate the sample size for the multiple regression logistic analysis; the following results were obtained: 129 patients were needed based on an effect size (f2) of 0.15, an α level of 0.05, a power of 0.95, and several predictors of 4 [26].

The normality of all data was assessed using the Shapiro–Wilk test, and equal variances were evaluated using the F-test when necessary. We assessed the differences between groups for normally and non-normally distributed data using the student’s *t*-test or Welch’s *t*-test and the Wilcoxon test or Pearson’s chi-square test, respectively. We identified the factors affecting PA at 1 year postoperatively using multivariate logistic regression. The PA at 1 year postoperatively was the dependent variable; the variables that were significantly different between the two groups from the univariate analysis were extracted as independent variables. Finally, the cutoff value of the preoperative CSA of the VM required for the PA to exceed the points at 1 year postoperatively was calculated using the receiver operating characteristic (ROC) curve. All statistical analyses were performed using SPSS Statistics (version 27.0.0.0; IBM, Armonk, NY, USA); the statistical significance was set at *p* < 0.05.

## 3. Results

**Figure 3:**
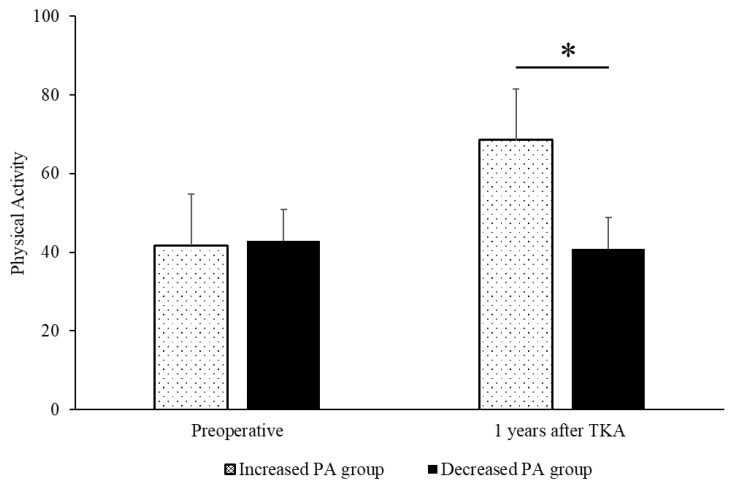
Changes in physical activity before TKA and at 1 year after TKA. There are significant differences between groups (* *p* < 0.001). TKA, total knee arthroplasty.

Table 1 presents patients’ demographic data. Of the patients included in this study, 30 in the decreased PA group had a bigger decrease in postoperative PA than in preoperative PA at 1 year after TKA. The increased PA group had a significant increase in PA 1 year after TKA compared with the decreased PA group (increased PA: 68.6 ± 14.3 points; decreased PA: 40.8 ± 7.7 points; *p* < 0.001) (Figure 3).

**Figure 4:**
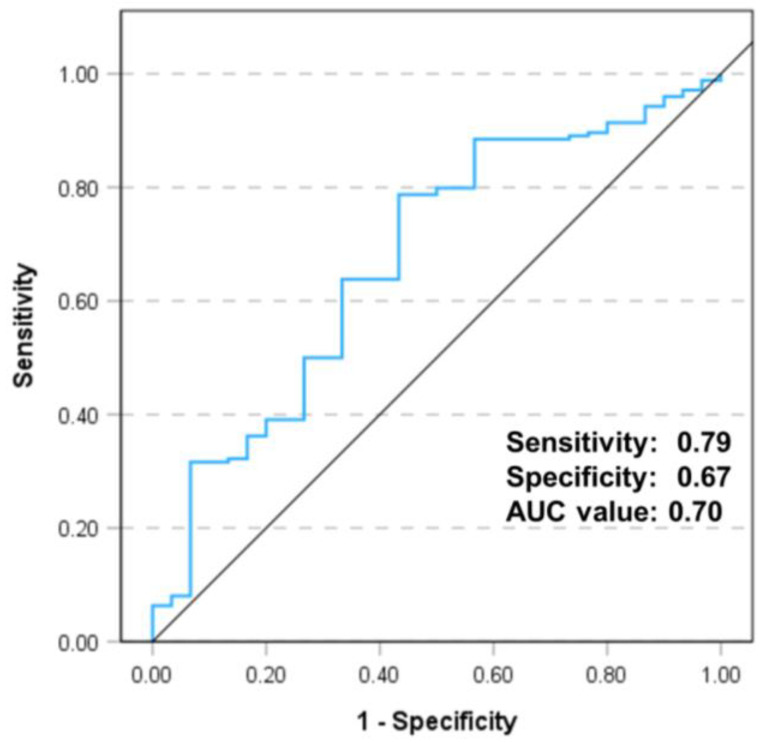
Calculation of cutoff values for preoperative-side and operative-side CSA of VM. The receiver operating characteristic curve demonstrates an area under the curve of 0.70 for increased and decreased 1-year postoperative PA. The optimal threshold to predict PA at 1 year postoperatively was the operative-side CSA of the VM at a preoperative angle of 10.2 cm^2^ (sensitivity: 0.79; specificity: 0.67). CSA, cross-sectional area; VM, vastus medialis; PA, physical activity; AUC, area under the curve.

The non-operative-side quadriceps strength, KOOS Sport/Rec scores (Table 2), operative-side CSA of the VM, and CTV of the VL (Table 3) showed significant differences using the univariate analysis. Multivariate logistic regression analysis (Table 4) showed that the non-operative-side quadriceps strength (odds ratio (OR): 1.011; 95% confidence interval (CI): 1.004–1.018; *p* = 0.001); KOOS sport/rec (OR: 1.037; 95% CI: 1.003–1.073; *p* = 0.033); operative-side CSA of VM (OR: 1.544; 95% CI: 1.124–2.119; *p* = 0.007), and operative-side CTV of VL (OR: 1.222; 95% CI: 1.063–1.404; *p* = 0.005) were significantly associated with sufficient PA at 1 year after TKA. The ROC curve showed that 10.2 cm^2^ was the cutoff value of the operative-side CSA of the VM for predicting the achieved PA at 1 year postoperatively (sensitivity: 0.79; specificity: 0.67); the area under the curve was 0.70 (Figure 4).

## 4. Discussion

This study identified several factors associated with the preoperative risk of decreased PA at 1 year after TKA. The results partially supported the study hypothesis that preoperative strength of quadriceps and operative-side CSA and CTV of the quadriceps would decrease, and postoperative PA would decrease in such cases. The clinical implication of this study was that an increase in the preoperative quantity of VM and quality of VL could improve PA after TKA.

The findings indicated that patients with decreased non-operative-side quadriceps strength had higher odds of no improvement in PA at 1 year after TKA. Consistent with previous studies [8,13,27], we confirmed that preoperative non-operative-side quadriceps strength was associated with postoperative function. In addition, previous studies reported that preoperative contralateral quadriceps strength predicted PA, stair-climbing ability, and walking speed after TKA [13,14]. The contralateral leg often compensates for the weakened operative side during rehabilitation and daily activities in patients undergoing TKA on the affected knee. Patients with stronger quadriceps muscles on the non-operative side were likely to support their body weight better during walking, stair climbing, and other physical tasks, contributing to more efficient recovery and enhanced mobility. These findings emphasized the importance of preoperative rehabilitation programs focusing on strengthening the affected limb and contralateral leg to optimize overall recovery.

This study showed that the preoperative CSA of the VM and CTV of the VL affected PA postoperatively. Moreover, the threshold for predicting improved PA at 1 year after TKA was a 10.2 cm^2^ preoperative CSA of the VM. A previous study reported that the VM muscle thickness in the severe KOA group was significantly smaller than that in the healthy group [28]. The quadriceps intramuscular adipose tissue, particularly in the VM, was significantly higher in the early KOA group than in healthy controls [29]. In age-related changes, an increase in quadriceps intramuscular adipose tissue occurred earlier than the loss of muscle quantity [16]. The VM, as part of the quadriceps group, was crucial for stabilizing the knee joint during movement, specifically in high-demand activities such as walking, stair-climbing, and squatting [30,31]. Therefore, an increase in intramuscular fat within the VM might have contributed to reduced knee stability and a subsequent decrease in PA. Previous studies have reported that resistance training could improve muscle quantity and quality [32,33]. In addition, preoperative high-intensity strength training rehabilitation improved lower limb muscle strength and knee ROM before surgery, resulting in a reduced length of stay at the hospital and faster physical and functional recovery after TKA [34,35]. From a clinical perspective, these findings highlighted the importance of preoperative rehabilitation strategies that future studies should consider incorporating to determine whether volume and fat infiltration in the muscle could improve after TKA. A previous study reported that high-velocity resistance training might be superior to low-velocity resistance training for enhancing muscle quality [36]. Therefore, implementing high-velocity resistance training during preoperative rehabilitation might lead to improvements not only in muscle volume but also in muscle quality.

This study found that patients with lower preoperative KOOS sport/rec scores had higher odds of no improvement in PA 1 year after TKA. The KOOS Sport/Rec subscale specifically evaluated the patient’s ability to engage in higher-level PA, such as squatting, running, and jumping, which demands significant knee function and strength [37,38]. Patients with lower preoperative KOOS Sport/Rec scores were likely to have greater limitations in these demanding activities, reflecting a more advanced knee function impairment. Some systematic reviews suggested that PA could recover within 3 months postoperatively and exceed preoperative levels at 6–12 months postoperatively [39,40]. However, most patients who underwent TKA retained less PA than age-matched controls throughout the 1 year postoperatively [39,40,41]. These limitations might be indicative of the affected knee’s condition and reflect a reduced overall capacity for PA, including the strength and coordination of the contralateral limb and overall conditioning. Consequently, individuals with lower preoperative KOOS Sport/Rec scores might face a more challenging postoperative recovery, particularly in terms of regaining mobility and achieving higher PA levels.

This study has some limitations. First, we exclusively assessed PA through subjective measures, specifically the KSS. The PA data obtained in this study differed from those measured by objective tools such as three-axis accelerometers or pedometers. Second, this was a single-center study, which might limit the generalizability of the findings to other populations or healthcare settings. Multicenter studies are required to confirm these results in different demographic and clinical settings. Finally, this study primarily focused on the quadriceps femoris muscles. In addition to the quadriceps femoris, other muscle groups, such as the hamstrings, could play a critical role in knee joint stability, mobility, and overall physical activity. The hamstrings act as antagonists to the quadriceps, providing dynamic stabilization to the knee joint by resisting anterior tibial translation and reducing stress on the reconstructed joint during walking and other activities. Although other muscle groups around the knee joint, such as the hamstrings, were not evaluated, they might have also contributed to postoperative PA and functional recovery.

## 5. Conclusions

The preoperative quadriceps strength on the non-operative side, the operative-side CSA of the VM and CTV of the VL, and preoperative KOOS Sport/Rec scores were significant predictors of achieving higher PA 1 year after TKA. These results suggested that preoperative quadriceps strength, muscle quantity, and quality, particularly of the VM, could play important roles in postoperative PA outcomes after TKA.

## Figures and Tables

**Table 1:** Patient demographic data.

Characteristics	N or Mean
Sex: males/females	49:155
Age at time of surgery (years)	73.5 (8.5)
Height (m)	155.0 (7.7)
Weight (kg)	61.8 (10.1)
BMI (kg/m^2^)	25.7 (3.9)
History of contralateral TKA, yes/no	48/156
Approach	
Subvastus	181 (88.7%)
Medial parapatellar	23 (11.3%)
Kellgren–Lawrence grade	
III	31 (15.2%)
IV	173 (84.8%)

BMI, body mass index; TKA, total knee arthroplasty.

**Table 2:** Comparison of preoperative parameters by increased or decreased 1-year PA.

Variable	Increased PA Group (n = 174)	Decreased PA Group (n = 30)	95% CI	*p* Value
Clinical parameters				
Gender: males/females	43:131	6:24	−0.11, 0.23	0.496
Age at time of surgery (years)	72.8 (8.7)	73.0 (7.2)	−3.17, 3.51	0.921
Height (m)	155.4 (7.8)	152. 9 (3.7)	−5.45, 0.50	0.103
Weight (kg)	62.1 (9.6)	60.1 (10.3)	−5.96, 1.97	0.322
BMI (kg/m^2^)	25.7 (3.6)	25.7 (4.0)	−0.11, 0.23	0.948
History of contralateral TKA	42	6	−0.21, 0.13	0.624
10 m gait time (s)	13.1 (5.2)	13.5 (5.2)	−1.64, 2.51	0.672
Operative side				
Knee flexion range of motion (°)	110.7 (16.2)	108.0 (14.4)	−8.95, 3.51	0.390
Knee extension range of motion (°)	−7.9 (6.5)	−8.2 (7.1)	−2.87, 2.31	0.831
Quadriceps strength (N)	169.9 (69.7)	146.7 (53.7)	−49.46, 3.27	0.086
One-leg standing time (s)	22.0 (17.0)	18.3 (16.2)	−10.32, 2.83	0.263
Knee pain	4.0 (1.5)	3.4 (1.9)	−1.23, 0.01	0.055
FTA	187.6 (5.4)	186.2 (5.3)	−3.53, 0.68	0.177
Non-operative side				
Knee flexion range of motion (°)	129.0 (12.5)	129.5 (11.5)	−4.34, 5.29	0.845
Knee extension range of motion (°)	−2.6 (4.8)	−1.3 (3.5)	−0.52, 3.09	0.163
Quadriceps strength (N)	213.2 (74.4)	154.4 (68.3)	−87.49, −30.17	<0.001
One-leg standing time (s)	31.4 (23.3)	33.5 (24.9)	−7.79, 11.92	0.657
Knee pain	1.5 (1.8)	1.8 (2.1)	−0.57, 1.08	0.482
FTA	176.8 (1.4)	176.9 (1.5)	−0.21, 0.57	0.654
PROMs				
KOOS symptoms	48.9 (19.0)	49.5 (16.5)	−6.72, 7.81	0.882
KOOS pain	46.9 (19.6)	41.5 (19.1)	−13.05, 0.26	0.164
KOOS ADL	52.1 (20.3)	50.2 (18.4)	−9.69, 5.96	0.639
KOOS Sport/Rec	22.1 (16.9)	13.8 (11.6)	−14.72, −2.04	0.010
KOOS QOL	26.8 (17.7)	26.0 (19.7)	−7.90, 6.15	0.806

PA, physical activity; BMI, body mass index; TKA, total knee arthroplasty; FTA, femorotibial angle; PROMs, patient-reported outcome measures; KOOS, knee injury and osteoarthritis outcome score; ADL, activities of daily living; QOL, quality of daily life; Sport/Rec, sports and recreation function; CI, confidence interval.

**Table 3:** Comparison of preoperative parameters by sufficient or insufficient 1-year PA.

Variable	Increased PA Group (n = 174)	Decreased PA Group (n = 30)	95% CI	*p* Value
Operative side				
Cross-sectional area				
Vastus medialis (cm^2^)	11.6 (1.8)	10.4 (1.5)	−1.53, −0.30	<0.001
Vastus lateralis (cm^2^)	19.2 (1.1)	19.1 (1.0)	−0.55, 0.33	0.619
Vastus intermedius (cm^2^)	17.1 (1.1)	17.0 (1.0)	−0.79, 1.65	0.491
Rectus femoris (cm^2^)	6.4 (0.7)	6.5 (0.5)	−0.21, 0.31	0.384
Computed tomography values				
Vastus medialis (HU)	44.5 (2.9)	43.5 (2.9)	−2.19, 0.07	0.065
Vastus lateralis (HU)	45.9 (3.0)	43.9 (3.3)	−3.23, −0.84	<0.001
Vastus intermedius (HU)	47.4 (3.3)	47.8 (3.6)	−0.79, 1.65	0.491
Rectus femoris (HU)	47.5 (3.3)	48.0 (3.1)	−0.71, 1.83	0.384
Non-operative side				
Cross-sectional area				
Vastus medialis (cm^2^)	11.9 (1.0)	12.0 (1.1)	−0.34, 0.46	0.761
Vastus lateralis (cm^2^)	21.9 (1.6)	21.9 (1.7)	−0.63, 0.61	0.979
Vastus intermedius (cm^2^)	17.3 (0.8)	17.3 (0.8)	−0.29, 0.32	0.923
Rectus femoris (cm^2^)	7.0 (0.8)	7.0 (0.9)	−0.29, 0.31	0.945
Computed tomography values				
Vastus medialis (HU)	51.3 (1.7)	51.3 (2.2)	−0.62, 0.80	0.801
Vastus lateralis (HU)	51.4 (1.8)	51.3 (2.1)	−0.76, 0.68	0.915
Vastus intermedius (HU)	51.0 (2.5)	51.2 (2.9)	−0.75, 1.21	0.646
Rectus femoris (HU)	50.6 (1.8)	50.5 (1.8)	−0.77, 0.65	0.863

CI, confidence interval; PA, physical activity; HU, Hounsfield unit.

**Table 4:** Preoperative predict factors of increased PA 1 year after primary TKA.

Variable	Odds Ratio	*p* Value	95% Confidence Interval	
			Lower	Upper
Non-operative-side quadriceps strength (N)	1.011	0.001	1.004	1.018
KOOS Sport/Rec	1.037	0.033	1.003	1.073
CSA vastus medialis (operative side)	1.544	0.007	1.124	2.119
CTV vastus lateralis (operative side)	1.222	0.005	1.063	1.404

KOOS, knee injury and osteoarthritis outcome score; CSA, cross-sectional area; CTV, computed tomography attenuation values; Sport/Rec, function in sports and recreation.

## Data Availability

The raw data supporting the conclusions of this article will be made available by the authors upon request.
